# MiR-206 regulates the progression of osteoporosis via targeting HDAC4

**DOI:** 10.1186/s40001-021-00480-3

**Published:** 2021-01-18

**Authors:** Zhiyuan Lu, Dawei Wang, Xuming Wang, Jilong Zou, Jiabing Sun, Zhenggang Bi

**Affiliations:** Department of Orthopedic Surgery, The First Affiliated Hospital of Harbin Medical University, No.23, Youzheng Street, Harbin, 150001 Heilongjiang China

**Keywords:** MiR-206, Osteoporosis, Cell proliferation, Cell apoptosis

## Abstract

**Background:**

More and more studies have confirmed that miRNAs play an important role in maintaining bone remodeling and bone metabolism. This study investigated the expression level of miR-206 in the serum of osteoporosis (OP) patients and explored the effect and mechanism of miR-206 on the occurrence and development of osteoporosis.

**Methods:**

120 postmenopausal women were recruited, including 63 cases with OP and 57 women without OP. The levels of miR-206 were determined by qRT-PCR technology. Spearman correlation coefficient was used to evaluate the correlation of miR-206 with bone mineral density (BMD). An ROC curve was used to evaluate the diagnostic value of miR-206 in osteoporosis. The effects of miR-206 on cell proliferation and cell apoptosis of hFOBs were measured by CCK-8 assay and flow cytometry, respectively. Luciferase reporter gene assay was used to confirm the interaction of miR-206 and the 3′UTR of HDAC4.

**Results:**

Serum miR-206 had low expression level in osteoporosis patient group compared with control group. The expression level of serum miR-206 had diagnostic value for osteoporosis, and the serum miR-206 levels were positively correlated with BMD. The down-regulated miR-206 could inhibit cell proliferation and promote cell apoptosis. Luciferase analysis indicated that HDAC4 was the target gene of miR-206.

**Conclusions:**

MiR-206 could be used as a new potential diagnostic biomarker for osteoporosis, and in in vitro cell experiments, miR-206 may regulate osteoblast cell proliferation and apoptosis by targeting HDAC4.

## Introduction

Osteoporosis (OP) is currently the most common bone disease recognized in the world. It is characterized by decreased bone mass, decreased bone strength, and bone tissue microstructure degradation [1]. The bone mass of a healthy adult reaches its peak during puberty and continues until the next bone remodeling cycle begins. The bones in the human body are constantly undergoing bone remodeling throughout their lives, which is the process of bone resorption by osteoclasts to damaged bone tissue and new bone formation by osteoblasts [1–3]. OP, a systemic metabolic bone disease, whose development mechanism is that the dynamic balance of bone remodeling is broken, which makes bone resorption far more than bone formation, and then systemic bone pain and various bones complications of the disease appeared. How is this dynamic equilibrium broken? Common causes include female estrogen deficiency, decreased parathyroid hormone levels, elevated glucocorticoid levels, and abnormal growth hormone levels [4]. Studies have found that more than 50% of osteoporotic fractures in women over 50 are due to an estimated 20% loss of bone density within 5 years after menopause [5, 6]. Therefore, to explore the pathogenesis of OP and find effective ways to prevent and treat OP is the foothold that should be paid more attention to at present.

MicroRNAs are small, non-coding single-stranded RNAs that regulate gene expression by blocking protein translation. Many miRNAs regulate various pathophysiological events, including hematopoietic function [7], organogenesis [8], cell proliferation and cell apoptosis [9], and tumorigenesis and progression [10]. MiRNAs are involved in bone cell differentiation, bone development, and bone disease. In the past few decades, a large number of miRNAs have been clearly found to be involved in the regulation of bone remodeling, including bone resorption and bone formation. The role of miRNAs in the growth and differentiation of osteoclasts and osteoblasts has been extensively studied [11]. Cao et al. conducted a preliminary study on miRNA biomarkers in circulating monocytes of patients with postmenopausal OP and found that the expression of miR-422a was down-regulated in the serum of OP patients, thus confirmed that miR-422a was a specific cell marker of postmenopausal OP [12]. MiR-206, previously viewed as a muscle-specific miRNA, has been reported to play a key role in osteosarcoma through regulating osteosarcoma cell viability and apoptosis [13]. Inose et al. reported that miR-206 regulated osteoblast differentiation by targeting connexin 43 (Cx43) in C2C12 cells [14]. Moreover, in the current study, miR-206 is identified to be significantly down-regulated in osteoporosis mouse model, indicating its potential role in the development of OP [15]. But the role and clinical value of miR-206 in OP is poorly understood.

Therefore, in the present study, in order to explore the clinical value of miR-206 in OP, the expression levels of serum miR-206 in postmenopausal OP patients and non-OP patients were measured, and the diagnostic value of miR-206 in OP was evaluated. In addition, its role in osteoblast cell proliferation and apoptosis was further explored in vitro.

## Materials and methods

### Study population and sample collection

This study protocol has been approved by the Clinical Research Ethics Committee of the First Affiliated Hospital of Harbin Medical University, and the recruited personnel have signed written informed consent.

A total of 120 postmenopausal women were recruited, including 63 postmenopausal women with OP as OP patient group and 57 postmenopausal women without OP as control group. Participants were selected based on the following conditions: (1) consent to participate in the study and informed consent, (2) no history of hormone replacement therapy, (3) no chronic malignant disease or genetic disease, and (4) no history of smoking or drinking.

### Cell culture

The main function of Human fetal osteoblast cell lines (hFOBs) is to produce bone matrix, secrete I collagen and non-collagen, and regulate bone absorption of osteoclast. The decrease of hFOBs viability caused by various factors can cause osteopenia and eventually lead to the occurrence of OP. Increasing the activity of hFOBs is one of the keys to prevent and treat metabolic bone diseases. Therefore, hFOBs was selected as the subject cell in this study [16, 17]. hFOBs (Chinese Academy of Medical Sciences, China) were cultured in Dulbecco Modified Eagle Medium (DMEM)/HAM F12 (Sigma-Aldrich, Dorset, UK) supplemented with 10% fetal bovine serum (FBS, Gibco), 1% triple antibiotic (penicillin, streptomycin, and amphotericin-B) (Solarbio), 0.6 mg/mL geneticin, and 2 mM l-glutamine (Gibco) at 37 ℃ temperature in a 5% CO_2_ and a 95% humidified atmosphere for 2 days of incubation.

### Cell transfection

MiR-206 inhibitor and a negative control (miR-NC) were purchased from Shanghai GenePharma Company. hFOBs were seeded onto 6-well plate at a density of 1 × 10^5^ cells per well, and after incubation for 24 h, cells were transfected with miR-NC and miR-206 inhibitor using lipofectamine2000 transfection reagent (Invitrogen, Carlsbad, CA, USA) to mimic OP condition by regulating miR-206 expression according to product specification.

### Total RNA extraction and qRT-PCR

Total RNA was isolated by TRIzol reagent (Invitrogen, Carlsbad, CA, USA) in accordance with the manufacturer’s instructions. RNA was then reverse transcribed into cDNA by the PrimeScript RT Reagent Kit (TaKaRa, Dalian, China). The qRT-PCR was conducted by SYBR Green Real-time PCR Master Mix (Toyobo Co. Ltd., Osaka, Japan) with the following conditions: 95 °C for 5 min and followed by 30 cycles of 95 °C for 30 s, 58–61 °C for 30 s, and 72 °C for 2 min. The U6 was used as an internal control, and the relative gene expression levels were calculated by the 2^−ΔΔ*Ct*^ method.

### Cell proliferation assay

Cell Counting Kit-8 (CCK-8) assay is based on the principle that mitochondrial dehydrogenases oxidize WST-8 to a formazan dye product. Cell proliferation of 24 h, 48 h, and 72 h was detected using the CCK-8 kit (Dojindo, Japan) according to the instructions. Briefly, cells were seeded in a density of 5 × 10^4^ cells/well onto the 96-well plates and cultured completely adherent to the wall, and then CCK-8 was added into the cells and incubated at 37 ℃ for 2 h. The absorbance was measured at 450 nm using a microplate reader (Thermo Fisher Scientific).

### Cell apoptosis assay

An Annexin V-FITC Apoptosis Detection kit (Keygen Biotechnology) was used for the quantitative analysis of the cell apoptosis using flow cytometry. Cells were seeded onto a 6-well plate at a density of 1 × 10^6^ cells per well. After 24 h of incubation, cells were harvest and washed with cold PBS buffer for 3 times. Next, cells were re-suspended with 0.5 mL of cold PBS to make the cell density is greater than 1 × 10^5^ cells, and then, 5 μL of Annexin V-FITC and 1 μL of PI staining solution were added into the cell suspension for another 15 min of incubation in the darkness. Finally, the cell apoptosis rates were measured by a flow cytometer (FACSCanto II flow cytometer, BD Biosciences).

### Luciferase reporter gene assay

The online program Target-scan 7.0 predicted that there was a binding site between miR-206 and HDAC4 3′UTR, so HDAC4 was speculated to be the potential target gene of miR-206. Then, we selected the luciferase reporter gene assay to verify this conjecture. The 3′-UTR of HDAC4 was cloned into PGL3 luciferase reporter gene vector to build wild type (WT) and mutant type (MT) of HDAC4 3′-UTR luciferase report vectors. Then the cells were co-transfected with the WT-HDAC4 or MUT-HDAC4 and miR-206 inhibitor or miR-NC. After incubation for 48 h, the luciferase activity of each group was detected in strict accordance with the instructions of the dual-luciferase reporting system (Promega, INC., USA). The fluorescent intensity of renal cells was used as an internal reference.

### Statistical analysis

All statistical analyses in this paper were employed by SPSS 21.0 software (SPSS Inc., Chicago, IL) and GraphPad Prism 7 software (GraphPad Software, Inc., USA). Data are presented as mean ± standard deviation (SD), and the differences in two groups were performed by Student’s *t*-test or one-way ANOVA. The Spearman correlation coefficient was used to assess the correlation between continuous variables. *P*-values were considered statistically significant as follows: ***P < 0.001. Each experiment was conducted in triplicate.

## Results

### Clinical characteristics

As shown in Table 1, there were no significant differences in age and BMI between the OP patient group (postmenopausal women with OP) and the control group (postmenopausal women without OP). It was worth noting that the value of LSBMD, FNBMD, and THBMD in OP patient group was significantly lower than that in the control group (*P* < 0.001).

**Table 1:** Clinical data of the study population

Variables	All subjects (*N* = 120)		*P* value
	Control (*n* = 57)	OP patients (*n* = 63)	
Age (years)	50.58 ± 4.14	49.97 ± 4.20	0.425
BMI (kg/m^2^)	23.44 ± 3.17	22.76 ± 3.10	0.240
LS BMD (g/cm^2^)	0.91 ± 0.05	0.80 ± 0.04	< 0.001
FN BMD (g/cm^2^)	0.71 ± 0.04	0.64 ± 0.04	< 0.001
TH BMD (g/cm^2^)	0.75 ± .04	0.67 ± 0.03	< 0.001

Data are expressed as *n* or mean ± standard deviation

*OP* osteoporosis, *BMI* body mass index, *LS BMD* lumbar spine bone mineral density, *FN BMD* femoral neck bone mineral density, *TH BMD* total hip bone mineral density

### Expression level of miR-206 in OP patients

**Fig. 1:**
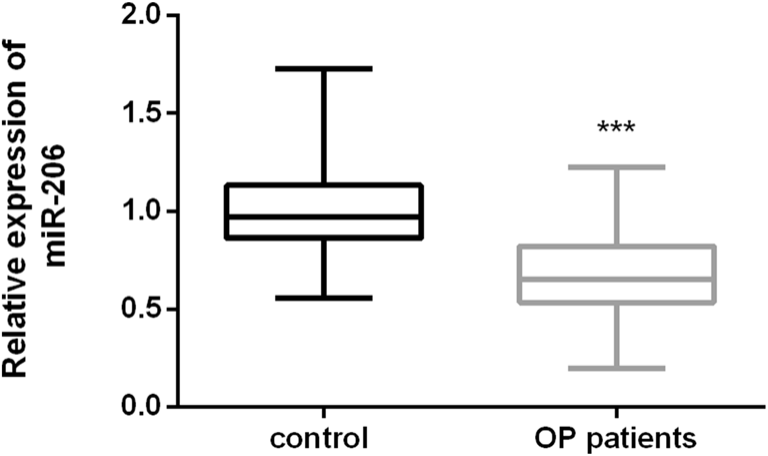
The expression level of miR-206 in the serum of OP patient group (postmenopausal women with OP) and control group (postmenopausal women without OP). The OP patient group had significantly low expression level of miR-206 (*P* < 0.001)

The serum expression levels of miR-206 in all participants were determined by qRT-PCR, and the results are shown in Fig. 1. The serum expression level of miR-206 in OP patient group was significantly lower than in the control group (*P* < 0.001), which suggested that the expression of miR-206 may be involved in bone formation and affect the progression of OP.

### Correlation analysis of miR-206 with LSBMD, FNBMD, and THBMD in OP patients

**Fig. 2:**
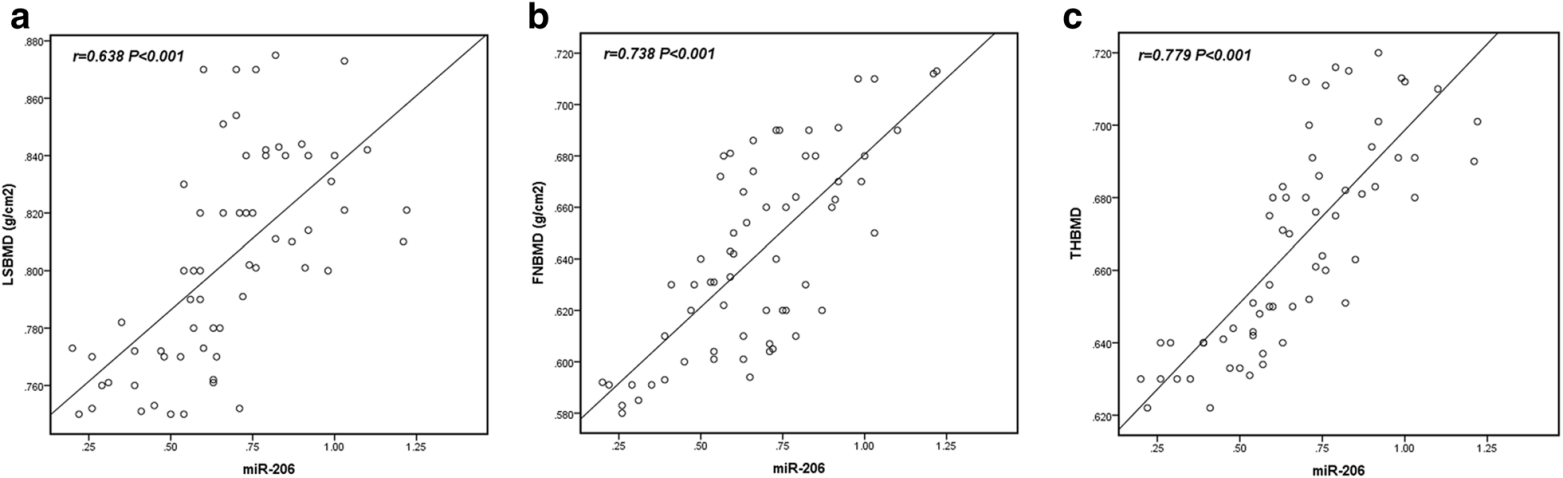
The correlation of serum miR-206 level with LSBMD (**a**), FNBMD (**b**) and THBMD (**c**) in OP patient group. Serum miR-206 levels were positively correlated with LSBMD (*r* = 0.638, *P* < 0.001), FNBMD (*r* = 0.738, *P* < 0.001), and THBMD (*r* = 0.779, *P* < 0.001) in OP patients

In the present study, the correlation of miR-206 expression with LSBMD, FNBMD, and THBMD was investigated and the results are shown in Fig. 2. The results showed that serum miR-206 in OP patients was positively correlated with LSBMD (*r* = 0.638, *P* < 0.001), FNBMD (*r* = 0.738, *P* < 0.001), and THBMD (*r* = 0.779, *P* < 0.001). The above results further indicated that miR-206 played an important role in the occurrence and progression of OP.

### ROC curve analysis

**Fig. 3:**
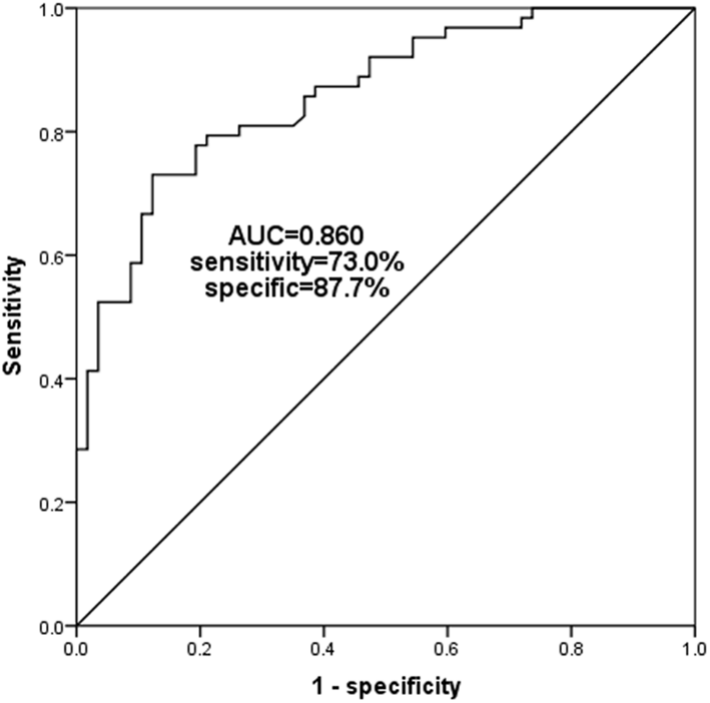
An ROC curve was established to evaluate the diagnostic ability of serum miR-206 in OP. The area under the curve (AUC) for miR-206 was 0.860, with a sensitivity of 73.0% and specificity of 87.7% at the cutoff value of 0.797

The ROC curve was established according to the expression level of serum miR-206 in the OP patient group and the control group. From the ROC curve in Fig. 3, the following conclusions can be drawn: the area under the curve (AUC) is 0.860, the sensitivity is 73.0%, and the specificity is 87.7%. Therefore, it can be inferred that the expression level of serum miR-206 can be used to distinguish patients with OP and without OP.

### Effects of miR-206 on hFOBs cell proliferation and apoptosis

**Fig. 4:**
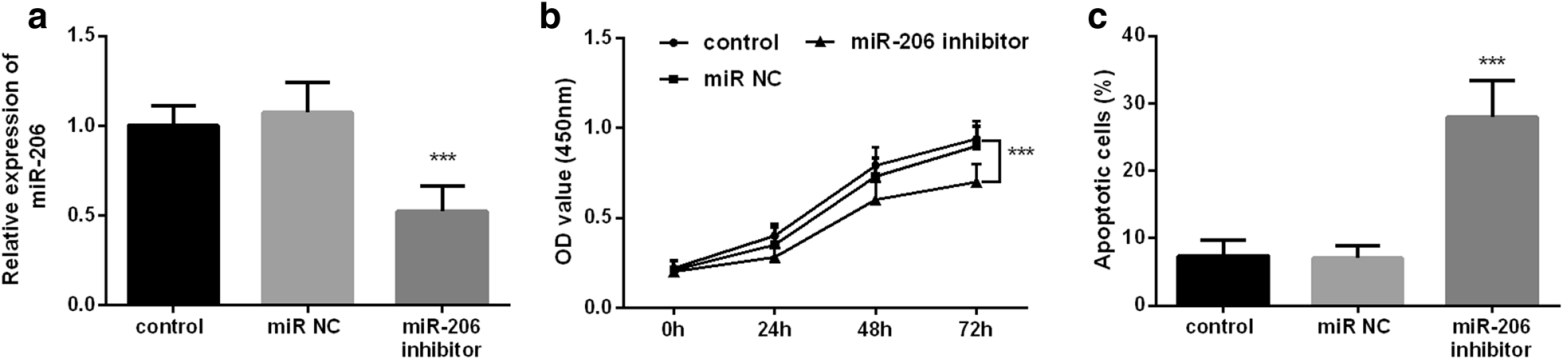
The relative expression level of miR-206 in cells transfected with different reagents and the effect of miR-206 on the proliferation and apoptosis of hFOBs. **a** Transfected with miR-206 inhibitor significantly decreased the expression level of miR-206 in hFOBs compared with other cell groups (*P* < 0.001). **b** The cell viability of hFOBs was significantly decreased in transfection with miR-206 inhibitor compared with other groups (*P* < 0.001). **c** The cell apoptosis of hFOBs was significantly increased in transfection with miR-206 inhibitor compared with other groups (*P* < 0.001)

Figure 4a shows the expression level of miR-206 in the cells after transfection with miR-206 inhibitor and miR-NC. It can be seen that the expression level of miR-206 in the miR-206 inhibitor group has been reduced significantly compared with the control group (*P* < 0.001), which can be used to simulate the low expression state of miR-206 in the serum of OP patients. The result of cell proliferation assay is shown in Fig. 4b; cell viability decreased significantly with the extension of cell incubation time after the down-regulation of miR-206 expression in comparison with the control group (*P* < 0.001). Cell apoptosis results showed that the apoptosis rates of miR-206 inhibitor group were significantly increased compared with the control group (Fig. 4c, *P* < 0.001). Based on the above experimental results, we have reason to believe that the down-regulated expression of miR-206 is not only detrimental to the cell viability of hFOBs but also promotes cell apoptosis.

### MiR-206 directly targets HDAC4 in hFOBs cells

**Fig. 5:**
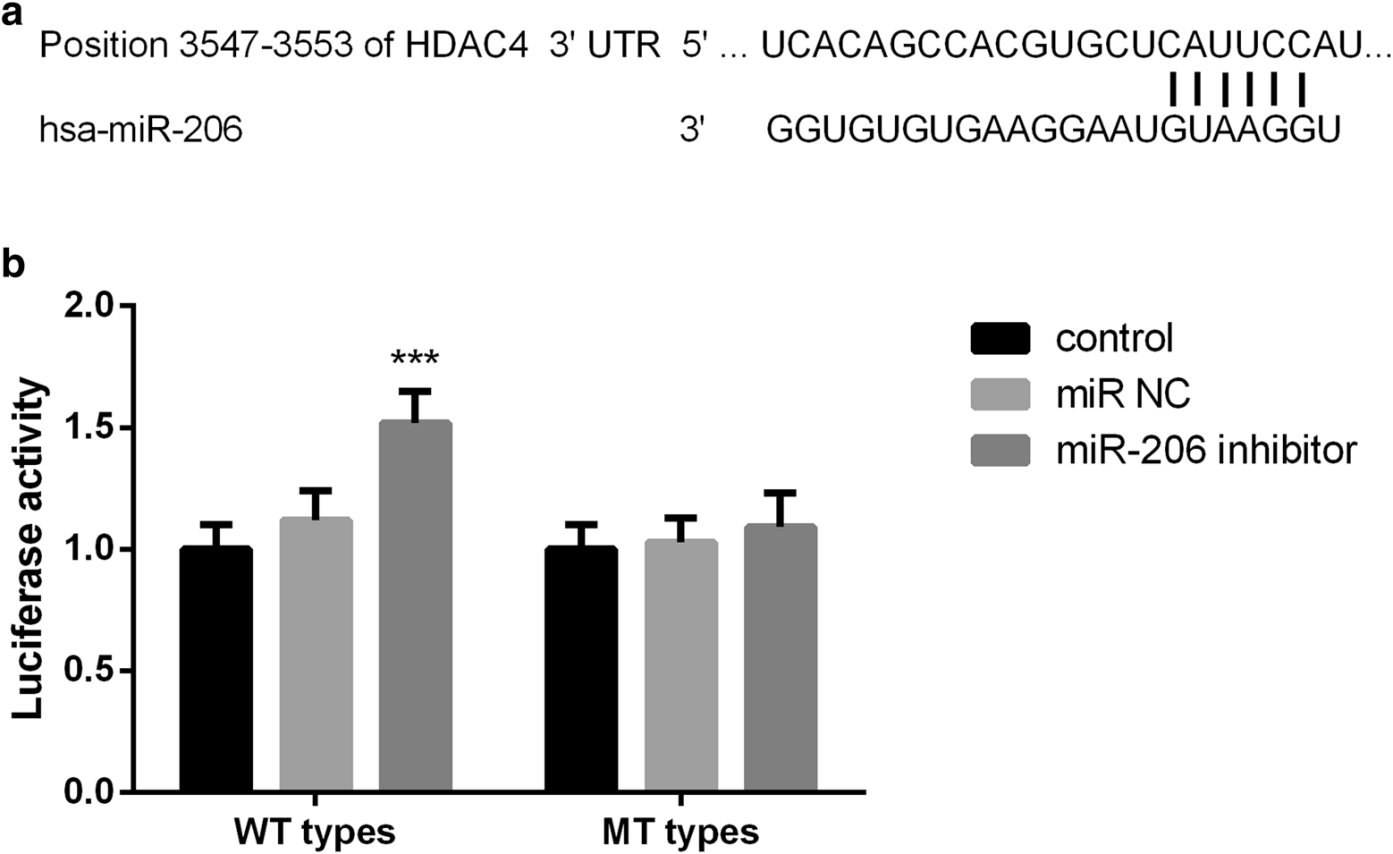
MiR-206 directly targets HDAC4 in hFOBs. **a** The binding site of miR-206 in HDAC4. **b** Cells transfected with miR-206 inhibitor significantly increased its luciferase activity. Besides, the luciferin activity of the mutant-type group was not affected by the transfection of miR-NC or miR-206 inhibitor (*P* > 0.05)

Target scan analysis results showed the binding sites of miR-206 in HDAC4 3′-UTR that are shown in Fig. 5a. Luciferase reporter gene assay results showed that transfected with miR-206 inhibitor significantly increased the luciferase activity in cells transfected with wide-type 3′-UTR of HDAC4 (*P* < 0.001). In addition, the transfection of miR-NC or miR-206 inhibitor did not affect the luciferase intensity of the MUT group.

## Discussion

Postmenopausal osteoporosis (PMOP) is one of the major manifestations of OP. In general, the dual processes of bone resorption and bone formation are tightly coupled to maintain a stable bone homeostasis state [18, 19]. For PMOP, postmenopausal women may experience a certain amount of bone loss, resulting in increased bone resorption and reduced bone formation. However, the exact mechanism behind PMOP is still unclear. Although osteoblasts and osteocyte apoptosis have been shown to play an important role in PMOP, there are still many issues that need to be explored and investigated [20, 21]. Osteoporosis-related fractures/brittleness and osteoarthritis are age-related diseases that have been increasing in incidence and prevalence in recent years due to an aging population [22, 23]. OP as a part of the natural aging process of the human body, its pathogenesis remains to be clarified. Current therapies, including hormone replacement therapy and immunotherapy, are associated with the risk of side effects. Therefore, it is vital to further understand the pathophysiological mechanism of OP for the development of new therapeutic methods.

MiR-206 is a member of the so-called myomiR family (i.e., miR-1, miR-133, and miR-206) and is widely believed to be a specific positive regulator of skeletal muscle differentiation [24, 25]. In this study, through the detection of the expression level of serum miR-206 in 120 postmenopausal women, we found that the expression level of serum miR-206 in the OP patient group was significantly lower than that in the control group, suggesting that miR-206 may play a certain role in the OP of postmenopausal women. In addition, the relevant indicators of OP, such as bone mineral density (BMD), were tested and the correlation between the expression level of miR-206 in serum and BMD was investigated. Finally, it was found that the expression level of miR-206 in OP patients was positively correlated with BMD, which further explained the correlation between miR-206 and OP. In the study of Elatta et al., it was found that serum miR-206 expression levels were significantly higher in patients with rheumatoid arthritis (RA) than in healthy controls (*P* < 0.001). At the same time, the serum inflammatory factors (IL-16 and IL-17) were significantly increased in RA patient group. Their study suggested the importance of miR-206 expression in RA patients as a potential new biomarker for bone loss/deformity, and revealed its synergistic role with pro-inflammatory cytokines, such as IL-16 and IL-17, in RA bone metabolism [26]. In another study, it was found that bone marrow mesenchymal stem cells (BMSCs) were isolated from ovariectomy (OVX) mice and normal mice for gene detection to screen out microRNAs that are most likely to participate in the mechanism of OP, including miR-206 [15]. All the above results supported our speculation on the role of miR-206 in the occurrence and development of OP. Moreover, through ROC curve analysis, we believed that miR-206 had the ability to distinguish postmenopausal women with OP from those without OP.

Dysfunction of osteoblasts is the most fundamental cause of OP. Therefore, it is necessary to explore the related functions of osteoblasts in revealing the mechanism of OP [27]. Subsequently, in this study, we investigated the effects of miR-206 on the proliferation and apoptosis of hFOBs. Cell transfection was used to down-regulate the expression of miR-206 in hFOBs, and the survival rate of cells transfected with miR-206-inhibitor was significantly reduced compared with untreated cells. In addition, the results of cell apoptosis assay were consistent with the results of cell proliferation inhibition assay, that is, after the expression of miR-206 was down-regulated, the cell apoptosis rate was significantly increased. The above results both indicated that when the expression level of miR-206 in hFOBs was low, it was not conducive to the proliferation of hFOBs and could lead to proliferation inhibition and apoptosis promotion. This result was also supported the observation about the low expression of miR-206 in serum of OP patients.

Studies have shown that the interaction of HDAC4, H3K9ac, and H3K27ac in OP is essential to maintain mineralized matrix synthesis and osteoclast regulation of the production of chemokines, which can protect bone tissue from the development of OP mediated by glucocorticoid overdose or estrogen deficiency [28]. Chen et al. found in their study that miR-19A-3P promoted the osteogenic differentiation of hMSCs by targeting HDAC4 and delayed the progression of osteoporosis [29]. At the end of this study, we preliminarily identified Histone Deacetylase 4 (HDAC4) as a potential target gene of miR-206 based on target scanning database. Based on the above findings, we selected luciferase reporter gene assay to determine whether HDAC4 is the target of miR-206. The results showed that luciferase activity was enhanced when miR-206 was down-regulated, suggesting that HDAC4 is the target gene of miR-206. Consistently, in a study of chronic constriction injury, HDAC4 has been proven to be the target gene of miR-206 [30].

In conclusion, this study confirmed that miR-206 was low expressed in serum of postmenopausal women with OP and had diagnostic value for OP. MiR-206 may be involved in the occurrence and development of OP by targeting HDAC4. The above results provided a basis for further elucidating the relationship between miR-206 and the occurrence and development of OP in postmenopausal women.

## Data Availability

The datasets used and/or analyzed during the current study are available from the corresponding author on reasonable request.

## Abbreviations

BMD: Bone mineral density
OP: Osteoporosis
hFOBs: Human fetal osteoblast cell lines
DMEM: Dulbecco Modified Eagle Medium
CCK-8: Cell Counting Kit-8
WT: Wild type
MT: Mutant type
SD: Standard deviation
AUC: Area under the curve
PMOP: Postmenopausal osteoporosis
RA: Rheumatoid arthritis
BMSCs: Bone marrow mesenchymal stem cells
OVX: Ovariectomy
HDAC4: Histone Deacetylase 4
